# Autopsies1Autopsies made in the laboratory of the New York College of Veterinary Surgeons.

**Published:** 1895-06

**Authors:** H. D. Gill


### AUTOPSIES.[1]

By H. D. Gill, V. S.

### Defective Development of the Central Nervous System in a Cat. (Brain, lxix.)

*Clinical Symptoms:* Paresis of both posterior extremities and right anterior extremity.

*Autopsy:* The marked feature was the fact that the right cerebellum was scarcely a third the size of the left. That this condition was the result of defective development was shown by the normal state of all the cerebellar tissue present. In addition it was noted that the right cerebral hemisphere, the right half of the pons and medulla, and the right crus cerebri were all somewhat smaller than the corresponding parts on the left side.

### Dermoid Cyst.

A tumor removed from the side of a mule, and reported to be of twenty years' growth, was presented to the museum by Dr. G. A. Waldron, of Tecumseh, Michigan. It proved to be a dermoid cyst about the size of a man's head. The cyst weighed six and one-half pounds and contained irregular masses of bone, cartilage and fibrous tissue, with a quantity of hair. It would have been interesting to know the size when first observed.

### Osteosarcoma in the Nose of an Ox.

The nasal cavities were filled by an irregularly rounded tumor, which was about nine inches by five inches in size. It seemed to have grown from the internal face of the external wall of the left antrum. It obliterated the left antrum and nearly filled the right. About three-fourths of the mass was made up of cancellous bone-tissue. The soft parts and the external surface of the tumor were irregularly lobular, and there seemed to be a capsule. There were one or two cysts which contained semi-gelatinous, light-colored, glairy fluid. Microscopically it was found to be made up of fibrous tissue, densely infiltrated with small spindle-shaped cells, having for their support a stroma

[1] Autopsies made in the laboratory of the New York College of Veterinary Surgeons.

derived from the fibrous bands which traversed the mass more abundantly in some places than others.

### Intussusception in a Dog.

*Clinical History:* The patient, a trick dog, the property of Huber's Museum of New York, was brought to the hospital January 17, 1895, with the following history: Three weeks before the dog had had distemper, but made a good recovery. On January 11th there began an attack of abdominal pain, diarrhœa, and bloody passages. January 17th examination showed a pulse of 100, temperature 103.4°, and a sausage-shaped tumor, about four inches long, situated in the abdomen posterior to the last rib and a little to the left of the median line. The dog was weak, in evident pain, was constantly thirsty, and had frequent bloody passages. Soon after arrival at the hospital all pain suddenly subsided, but the dog died the morning of the 19th.

*Autopsy:* A well-developed, somewhat emaciated "Ulmer" dog, lungs and heart normal. On opening the abdomen there was immediately apparent an invagination four and one-half inches long, occupying the colon about twenty-eight inches from the anus. There was a pea-sized perforation of the colon at a point near the lower end of the tumor. The coils of intestines were dilated, injected, and covered with flakes of fibrin. The peritoneal cavity contained about a pint of blood-stained serum of an offensive odor. The mesenteric glands were enlarged.

*Diagnosis:* Intussusception of colon, general peritonitis, perforation of intestine.

